# Simultaneous detection of reduced and oxidized forms of coenzyme Q10 in human cerebral spinal fluid as a potential marker of oxidative stress

**DOI:** 10.3164/jcbn.17-131

**Published:** 2018-06-08

**Authors:** Midori Nagase, Yorihiro Yamamoto, Jun Mitsui, Shoji Tsuji

**Affiliations:** 1 School of Bioscience and Biotechnology, Tokyo University of Technology, 1404-1 Katakura-cho, Hachioji, Tokyo 192-0982, Japan; 2 Department of Molecular Neurology, Graduate School of Medicine, The University of Tokyo, 7-3-1 Hongo, Bunkyo, Tokyo 113-8655, Japan; 3 Department of Molecular Neurology, Graduate School of Medicine, The University of Tokyo and International University of Health and Welfare, 4-3 Kouzunomori, Narita City, Chiba 286-8686, Japan

**Keywords:** coenzyme Q10, *tert*-butylhydroquinone, cerebral spinal fluid, column switching, electrochemical detection

## Abstract

The redox balance of coenzyme Q10 in human plasma is a good marker of oxidative stress because the reduced form of coenzyme Q10 (ubiquinol-10) is very sensitive to oxidation and is quantitatively converted to its oxidized form (ubiquinone-10). Here we describe an HPLC method for simultaneous detection of ubiquinol-10 and ubiquinone-10 in human cerebral spinal fluid to meet a recent demand for measuring local oxidative stress. Since the levels of coenzyme Q10 in human cerebral spinal fluid are less than 1/500 of those in human plasma, cerebral spinal fluid extracted with 2-propanol requires concentration for electrochemical detection. Using human plasma diluted 500-fold with physiological saline as a pseudo-cerebral spinal fluid, we found that addition of *tert*-butylhydroquinone was effective in preventing the oxidation of ubiquinol-10. The optimized *tert*-butylhydroquinone concentration in the extraction solvent was 20 µM. The addition of 20 µM ascorbic acid or co-addition of *tert*-butylhydroquinone and ascorbic acid (20 µM each) were also effective in preventing the oxidation of ubiquinol-10, but ascorbic acid alone gave poor reproducibility. Good within day reproducibility was observed, and day-to-day analytical variance was excellent.

## Introduction

Increases in oxidative stress have been suggested to contribute to aging and degenerative diseases such as heart attack, stroke, neurodegenerative diseases, diabetes, and cancer.^(1)^ Oxidative stress is defined as a disturbance in the pro-oxidant-antioxidant balance in favor of pro-oxidants.^(2)^ The redox balance of coenzyme Q10 (CoQ10) is a good marker of oxidative stress because its reduced form (CoQ10H_2_) is highly reactive with oxygen radicals and is converted to the oxidized form (CoQ10).^(1)^ In fact, the incubation of human plasma at 37°C under aerobic conditions in the presence of 5 µM cupric ion initially produces a decrease in vitamin C (VC), followed by a decrease in CoQ10H_2_ and the concomitant production of an equal amount of CoQ10.^(3)^ This observation indicates that the percentage of CoQ10 in total coenzyme Q10 (%CoQ10) represents a good marker of early stage oxidative stress. We therefore developed a simple and reliable method for the simultaneous electrochemical detection of plasma CoQ10H_2_ and CoQ10^(4)^ and applied the method to patients with various diseases. Significant increases in %CoQ10 were observed in patients with hepatitis,^(5)^ cirrhosis,^(5)^ hepatoma,^(5)^ juvenile fibromyalgia,^(6)^ amyotrophic lateral sclerosis (ALS),^(7)^ Parkinson disease,^(8)^ post cardiac arrest syndrome,^(9)^ and sepsis^(10)^ as compared to age-matched healthy controls. It is interesting that newborn babies have significantly higher plasma %CoQ10 than adults^(11)^ and %CoQ10 increases with age.^(12,13)^

Recently, the measurement of local oxidative stress is sought in order to gain a more precise understanding of the role of reactive oxygen species. For this purpose human cerebral spinal fluid (CSF) represents a good target compartment. However, the levels of total coenzyme Q10 (TQ10) in human CSF are less than 1/500 of those in human plasma. Therefore, CSF extracted with 2-propanol will require concentration prior to electrochemical detection. We prepared pseudo-CSF by preparing a 500 fold dilution of human plasma, which was used to optimize the analytical procedure.

Plasma VC was useful to prevent the oxidation of the reduced form of coenzyme Q10 in 2-propanol extracts (unpublished data). Although, CSF contains ~300 µM ascorbic acid, we found that the addition of *tert*-butylhydroquinone (TBHQ) was effective in preventing the oxidation of the reduced coenzyme Q10 during the extraction and concentration steps. We found the optimal TBHQ concentration to be 20 µM. A column switching system was useful in avoiding interference by TBHQ in the detection of vitamin E (VE), CoQ10H_2_, and CoQ10. Good within-day reproducibility was observed and day-to-day variance of the analytical method was excellent. Finally, we demonstrated that our method is applicable to human CSF.

## Materials and Methods

### Reagents, pseudo-CSF and human CSF

CoQ10H_2_ and CoQ10 were generous gifts from Kaneka (Osaka, Japan). TBHQ, 2-propanol (IPA), sodium perchlorate (NaClO_4_) and other chemicals were of the highest grade commercially available. We prepared pseudo-CSF by diluting human plasma with 499 volumes of 0.9% physiological saline. Human CSF was obtained from the Department of Neurology, Graduate School of Medicine, The University of Tokyo. Human CSF samples were obtained after written informed consent was obtained from participants and the research project was approved by the institutional review board of the University of Tokyo, School of Medicine.

### Optimized analytical procedure

Three hundred µl of Pseudo-CSF (or CSF) and 1.2 ml of 20 µM TBHQ in IPA were mixed in a 2.0 ml-polypropylene tube. After centrifugation at 3,000 rpm for 10 min at 4°C, 1.2 ml of supernatant was transferred into a 1.5 ml-LoBind tube (Eppendorf, Hamburg, Germany). The supernatant was dried under a stream of nitrogen gas, and the residue was admixed with 120 µl of IPA. Aliquots (60 µl) were injected into the HPLC system described below.

### HPLC system for simultaneous detection of CoQ10H_2_ and CoQ10

**Fig. 1:**
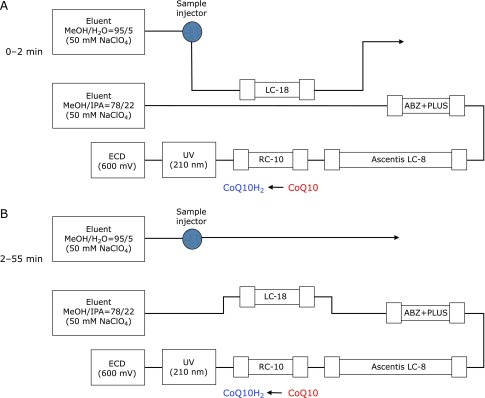
Column switching system for the analysis of CoQ10H_2_ and CoQ10 in human CSF.

In order to avoid interference by water-soluble antioxidants such as VC and TBHQ in the analysis of CoQ10H_2_ and CoQ10 using an octylsilyl column, a column switching method was employed (Fig. 1). Nanospace SI-2 systems (Shiseido, Tokyo, Japan) consisting of a 3,033 autosampler, two 3,011 pumps, a 3,004 column oven, a 3,011 high pressure valve, and a 3,005 electrochemical detector (ECD) (600 mV vs Ag/AgCl) were configured. A UV detector (210 nm; Model UV-970, Japan Spectroscopic, Tokyo, Japan) and an integrator (Model S-MC 21, Shiseido) were also used. A cleanup column (Supelcosil LC-18, 3 µm, 33 mm × 4.6 mm i.d.; Supelco Japan, Tokyo, Japan), two separation columns (Ascentis LC-8, 5 µm, 250 mm × 4.6 mm i.d. and Supelcosil ABZ + Plus, 3 µm, 33 mm × 4.6 mm i.d.; Supelco Japan), and a reduction column (RC-10, 15 mm × 4 mm i.d.; IRICA, Kyoto, Japan) were employed.

The mobile phase for the cleanup column was 50 mM NaClO_4_ in methanol/H_2_O (95/5, v/v) and was delivered at a flow rate of 0.4 ml/min. The mobile phase for the separation columns was 50 mM sodium NaClO_4_ in methanol/IPA (78/22, v/v) and was delivered at a flow rate of 0.8 ml/min. The columns were maintained at 20°C.

### Statistical analysis

Data presented are mean values and SD. Statistical analysis was performed with a Student’s *t* test.

## Results and Discussion

### Calibration curves

**Fig. 2:**
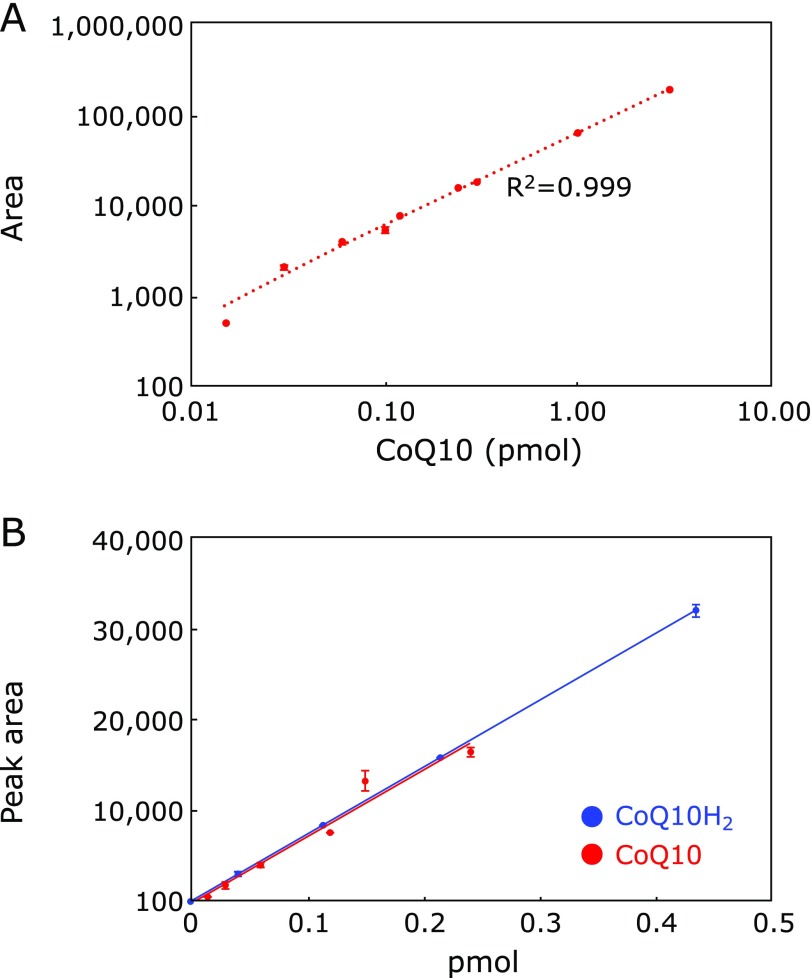
Calibration curves for CoQ10H_2_ and CoQ10.

Calibration curve for CoQ10 is shown in Fig. 2A. CoQ10 shows a good linear relationship over a range of 0.015–3 pmol, indicating that CoQ10 can be detected over a wide range. CoQ10 is not detectable with an ECD in oxidation mode. However, CoQ10 was converted to CoQ10H_2_ by the in line reduction column, thereby making the resulting CoQ10H_2_ detectable with the ECD. Almost identical slopes for CoQ10 and CoQ10H_2_ were observed (Fig. 2B), indicating that the reduction of CoQ10 to CoQ10H_2_ was quantitative.

### Separation of analytes from TBHQ and VC

**Fig. 3:**
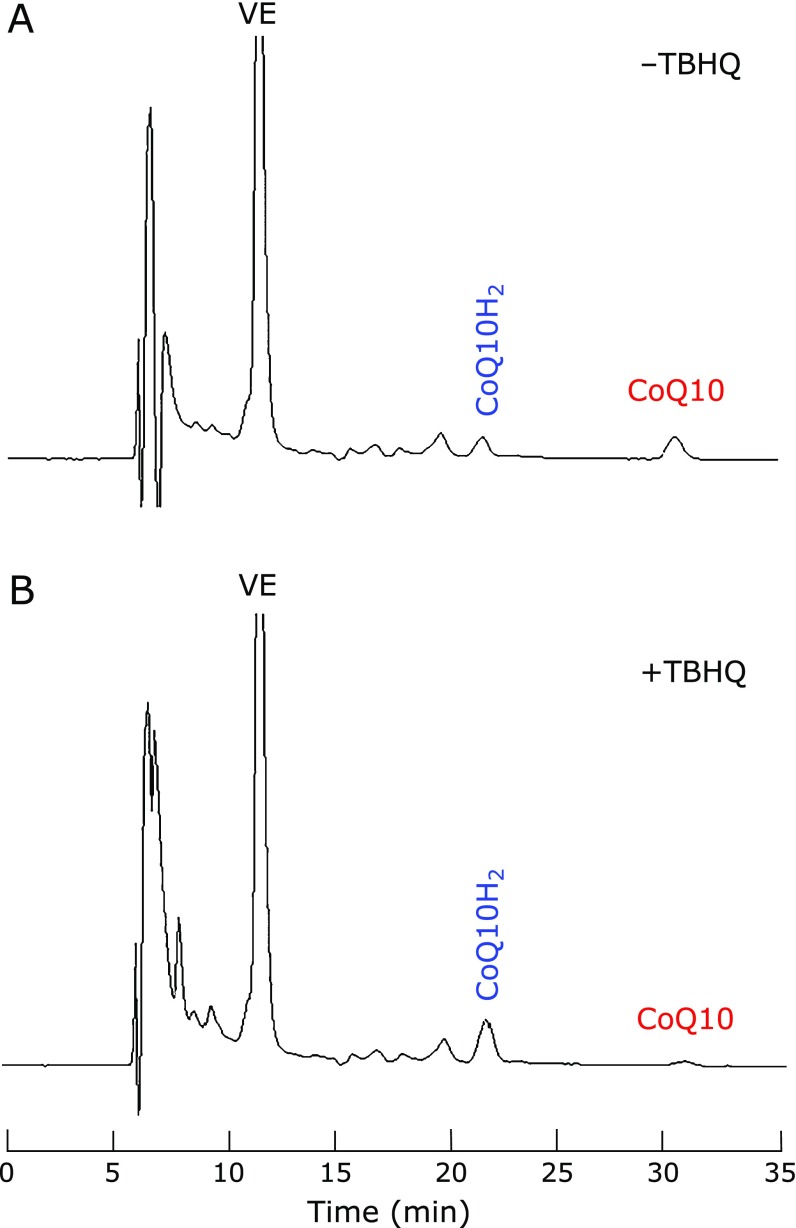
Typical HPLC chromatograms of IPA extracts of pseudo-CSF. Extraction was carried out without (A) and with (B) 20 µM TBHQ.

We set the column-switching time to 2 min (Fig. 1). For the first 2 min after the sample injection, analytes were introduced to the cleanup LC-18 column, which retains non-polar compounds such as VE, FC, CoQ10H_2_, and CoQ10, but not polar compounds such as VC and TBHQ. After 2 min, the cleanup column was connected to analytical columns and more non-polar mobile phase (IPA containing methanol) was delivered. In this condition, analytes eluted in the following order: VE, CoQ10H_2_, and CoQ10 as shown in the typical HPLC chromatograms of extract from pseudo-CSF (Fig. 3).

### Prevention of the CoQ10H_2_ oxidation by reductants

**Fig. 4:**
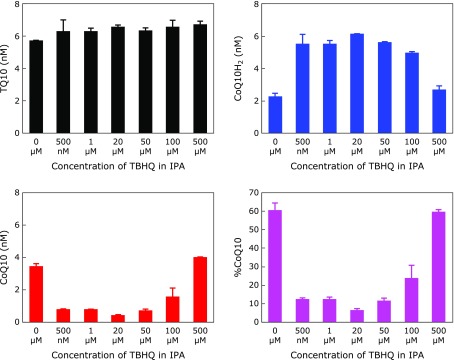
Effect of TBHQ concentrations in the extracting IPA on the detected levels of TQ10, CoQ10H_2_, and CoQ10, and %CoQ10 in pseudo-CSF.

Figure 3 also shows that the presence of 20 µM TBHQ in the extraction solvent (IPA) clearly inhibited the oxidation of CoQ10H_2_ to CoQ10. This is consistent with expectations as pseudo-CSF contained very low VC concentrations. The VC level in human plasma is ~30 µM, thus the VC concentration in pseudo-CSF is calculated to be 30/500 = 0.06 µM. Figure 4 shows the effect of the TBHQ concentration in the extraction solvent on the measurement of TQ10, CoQ10H_2_, and CoQ10 in pseudo-CSF. Levels of TQ10 were the same, suggesting that TQ10 was well recovered. However, the amount of detected CoQ10H_2_ increased with increasing TBHQ concentrations in the extraction solvent (up to 20 µM). Therefore, %CoQ10 was lowest at 20 µM TBHQ in the extraction solvent. Under this condition, the molar ratio of TBHQ to CoQ10H_2_ is calculated as 20 µM × 1,200 µl/(6 nM × 300 µl) = 13,000, indicating that sufficient amounts of TBHQ were present to inhibit the oxidation of CoQ10H_2_. However, more than 50 µM TBHQ in the extraction solvent appeared to induce autoxidation of TBHQ and consequently resulted in an increase of %CoQ10. Therefore, the optimal TBHQ concentration in the extraction solvent was deemed to be 20 µM.

**Fig. 5:**
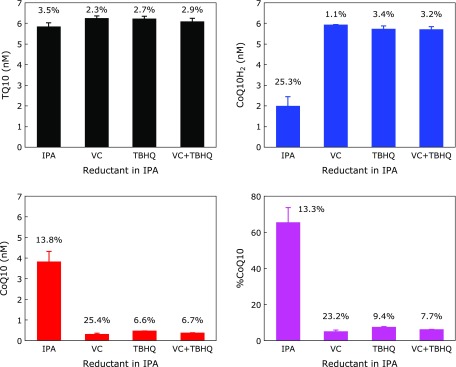
Effect of VC, TBHQ, and VC + TBHQ (20 µM each) in the extracting IPA on the detected levels of TQ10, CoQ10H_2_, and CoQ10, and %CoQ10 in pseudo-CSF. Numbers show the coefficients of variation (CV).

Next we compared TBHQ, VC, and TBHQ + VC as reductants (20 µM each) in the extraction solvent, and the results are shown in Fig. 5. The coefficients of variation (CV) for each condition are presented in the figure. There were no significant difference in the levels of TQ10, CoQ10H_2_, CoQ10 and %CoQ10 values in the presence of either the individual reductants or both reductants. However, poor reproducibility in CoQ10 detection was observed when VC was used as a reductant in the extraction solvent. When TBHQ was employed as the reductant, the reproducibility in CoQ10 detection was excellent. More importantly, the results in the presence of both VC and TBHQ were identical to those with TBHQ alone, indicating no interference with the detection of CoQ10H_2_ and CoQ10. Levels of VC in human CSF are reported to be about 200 µM,^(14)^ and we confirmed this observation. 200 µM VC in CSF corresponds to 50 µM VC in the extraction solvent. This may be sufficient to prevent CoQ10H_2_ oxidation during the extraction and concentration procedure. However, we chose to add 20 µM TBHQ to the extraction solvent to prepare for the case of minute VC levels being present in patients’ CSF. We would like to also emphasize that the addition of 20 µM TBHQ gave excellent reproducibility.

### Spiking and recovery

Table 1 shows the results of spiked 3.43 nM CoQ10H_2_ and 1.24 nM CoQ10 added to pseudo-CSF. The recovery of spiked CoQ10H_2_ and CoQ10 to pseudo-CSF were excellent (*n* = 3), as was TQ10 by extension.

### Reproducibility of the analysis

Table 2 shows the reproducibility of the detection of VE, CoQ10H_2_ and CoQ10 in pseudo-CSF (*n* = 3). The CV for VE, CoQ10H_2_, CoQ10, TQ10 and %CoQ10 were less than 10%, showing excellent within-day reproducibility. Table 3 shows the day-to-day variance of the analysis. The CVs for VE, CoQ10H_2_, CoQ10, TQ10 and %CoQ10 were less than 15%, indicating that the method is reliable.

### Application of the method to human CSF

**Fig. 6:**
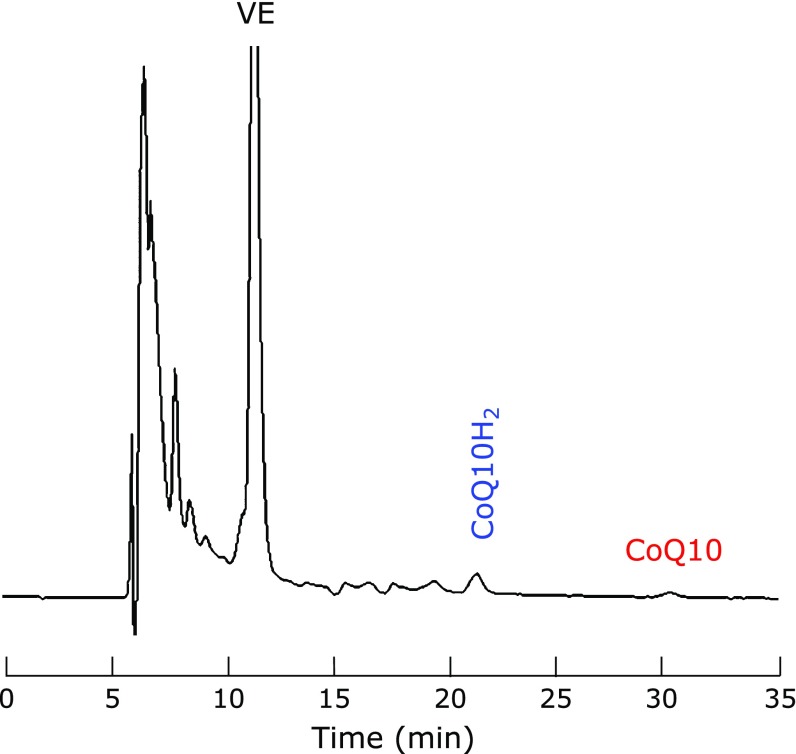
A typical HPLC chromatogram of an IPA extract of human CSF. Extraction was carried out with IPA containing 20 nM TBHQ.

Figure 6 shows a typical HPLC chromatogram of the IPA extract of human CSF. The obtained chromatogram was identical to that of pseudo-CSF (Fig. 3), suggesting that interference by the components in human CSF was not observed. Good reproducibility was also observed (Table 4). Thus, we concluded that the present method is useful for the evaluation of oxidative stress in human CSF.

A method for the simultaneous electrochemical detection of CoQ10H_2_ and CoQ10 was described. Since the levels of TQ10 in human CSF is ~2 nM, the IPA extract required concentrations prior to analysis. To prevent the oxidation of CoQ10H_2_ during extraction and concentration, 20 µM TBHQ was added to the extraction solvent (IPA). Good within-day and day-to-day reproducibility was observed and the analytical variance was excellent. We demonstrated our method is applicable to human CSF.

## Figures and Tables

**Table 1:** Recovery of spiked CoQ10H_2_, CoQ10, and TQ10 added to pseudo-CSF (*n* = 3)

	CoQ10H_2_ (nM)	CoQ10 (nM)	TQ10 (nM)
Pseudo-CSF	5.74 ± 0.62	1.08 ± 0.45	6.82 ± 0.17
Additive	3.43	1.24	4.67
Spiked sample	9.64 ± 0.22	2.23 ± 0.27	11.87 ± 0.24
Calculated	9.17	2.32	11.49
Recovery (%)	105.1	96.2	103.3

**Table 2:** Reproducibilities of the analysis of VE, CoQ10H_2_, CoQ10, TQ10 and %CoQ10 in pseudo-CSF (*n* = 3)

	VE (nM)	CoQ10H_2_ (nM)	CoQ10 (nM)	TQ10 (nM)	%CoQ
Average ± SD	59.2 ± 1.1	5.90 ± 0.06	0.44 ± 0.03	6.3 ± 0.04	7.0 ± 0.56
CV (%)	1.9	1.1	7.6	0.6	8

**Table 3:** Day-to-day variance of the analysis of VE, CoQ10H_2_, CoQ10, TQ10 and %CoQ10 in pseudo-CSF

	VE (nM)	CoQ10H_2_ (nM)	CoQ10 (nM)	TQ10 (nM)	%CoQ
Average ± SD	59.7 ± 0.68	5.73 ± 0.21	0.49 ± 0.05	6.2 ± 0.1	7.9 ± 0.9
CV (%)	1.1	3.6	9.3	1.6	11.5

**Table 4:** Reproducibilities of the analysis of VE, CoQ10H_2_, CoQ10, TQ10 and %CoQ10 in human CSF (*n* = 3)

	VE (nM)	CoQ10H_2_ (nM)	CoQ10 (nM)	TQ10 (nM)	%CoQ
Average ± SD	48.7 ± 5.2	1.67 ± 0.22	0.49 ± 0.02	2.16 ± 0.22	22.8 ± 2.2
CV (%)	10.8	13.1	3.4	10.3	9.5

## Abbreviations

CoQ10: oxidized form of coenzyme Q10
CoQ10H_2_: ubiquinol-10, reduced form of coenzyme Q10
CSF: cerebral spinal fluid
CV: coefficient of variation
ECD: electrochemical detector
IPA: 2-propanol
NaClO_4_: sodium perchlorate
TBHQ: *tert*-butylhydroquinone
TQ10: total coenzyme Q10
VC: vitamin C
VE: vitamin E
